# Self-reported symptom occurrence and distress, and psychological well-being after liver transplantation – a descriptive cross-sectional study of Danish recipients

**DOI:** 10.3389/fpsyg.2024.1354706

**Published:** 2024-03-13

**Authors:** Kristine Elberg Dengsø, Andreas Dehlbæk Knudsen, Dina Leth Møller, Anna Forsberg, Susanne Dam Nielsen, Jens Hillingsø

**Affiliations:** ^1^Department of Surgical Gastroenterology, Rigshospitalet, University of Copenhagen, Copenhagen, Denmark; ^2^Viro-Immunology Research Unit, Department of Infectious Diseases, Rigshospitalet, University of Copenhagen, Copenhagen, Denmark; ^3^Department of Health Sciences, Lund University, Lund, Sweden; ^4^Department of Thoracic Surgery, Skåne University Hospital, Malmö, Sweden

**Keywords:** liver transplantation, symptom distress, well-being, liver transplant recipients, survey

## Abstract

**Introduction:**

Symptom distress and impaired psychological well-being after liver transplantation may lead to limitations in everyday activities and lowered health-related quality of life. The aim of this nationwide, descriptive, and cross-sectional study was to explore self-reported symptom occurrence and distress, among Danish liver transplant recipients, and their association with self-reported psychological well-being as well as demographic, and clinical characteristics.

**Methods:**

Liver transplant recipients transplanted from 1990 to 2022 were included. All recipients were asked to complete the Organ Transplant Symptom and Wellbeing instruments consisting of two instruments measuring self-reported symptom occurrence and distress, respectively, as well as self-reported psychological well-being by the Psychological General well-being instrument.

**Results:**

Of 511 invited recipients 238 responded: 116 women and 122 men with a median post-transplant follow-up of 7.5 years (IQR 3.6–14.2 years). The most common single symptoms reported were decreased libido (18%), diarrhea (10%), and headache (8%). Sleep problems were the most common transplant-specific domain. 41% of the recipients reported poor psychological well-being, especially those who had undergone transplantation within the last 5 years, women, and younger recipients.

**Discussion:**

In the interest of equity, the fact that women reported a higher level of symptom distress than men requires attention. Research on symptom management support is warranted with interventions focusing on how to alleviate symptom distress, which might increase long-term survival, which has not improved in recent decades.

## Introduction

Liver transplantation (LT) leaves the recipient with a new chronic condition that necessitates specialized lifelong follow-up and immunosuppressive medication to prevent graft rejection. Lifelong follow-up should support the recipient to adapt to the new situation, including maintaining social relationships and being aware of bodily signs and symptoms (Moayed et al., 2018). Living with this new chronic condition requires extensive self-management (World Health Organization, 2002; Ko et al., 2019). Self-management is defined as the individual’s ability to manage symptoms, treatment, and lifestyle changes, consequences of the chronic condition, and encompasses a transformation where the organ transplantation is seen as a chronic condition (World Health Organization, 2002). Recipients’ self-management after LT consists of active participation in mandatory lifelong follow-up care, and self-monitoring of symptoms and signs that can potentially lead to rejection or be a sign of infection. Graft survival depends on the recipient’s self-management and adherence to the lifelong follow-up in the outpatient clinic (Moayed et al., 2018; Ko et al., 2019). For example, it has been found that recipients consider this regime difficult to implement, e.g., dietary guidelines, and often underestimate the strictness of immunosuppressive medication intake (Vanhoof et al., 2018). Even though the recipient’s quality of life (QoL) is increased after LT potential surgical complications and side effects caused by the lifelong immunosuppressive medication might negatively affect recipients (Dew et al., 1997), in terms of symptom occurrence and the level of the perceived symptom distress and poor psychological well-being (Drent et al., 2008, 2009; Kugler et al., 2009). Depression has been found to increase the risk of post-transplant mortality (Dew et al., 2015). Symptom distress is pivotal as this measures the recipients’ symptom experience. It is a significant post-transplant outcome that may affect the liver recipients’ self-management. An association between side effects from immunosuppressive medication, symptom experience and non-adherence has been found after solid organ transplantation as recipients may try to minimize the symptoms by decreasing the dosage (Drent et al., 2008, 2009; Kugler et al., 2009). Moreover, recipients’ psychological well-being might also be affected by existential challenges involved in being a transplant recipient and having received an organ from another person (Lundmark et al., 2016; Herzer et al., 2020).

The exploration of symptom occurrence, distress, and psychological well-being in relation to relevant sociodemographic characteristics may potentially facilitate target-oriented follow-up care and might help identify recipients who need tailored, person-centered self-management support. Research on heart recipients suggests that symptoms cause uncertainty, which might cause psychological distress, low self-efficacy, and impaired self-management (Almgren et al., 2017a,b). In liver recipients, self-management and psychological well-being are further affected by complications and the fairly extensive follow-up including monthly outpatient visits (Akbulut et al., 2021). Untreated symptoms may have a negative impact on recipients’ QoL and everyday life. This may place a further financial burden on the healthcare system due to an increased number of potentially avoidable outpatient visits (Almgren et al., 2017a,b). Therefore, the aim of the present study was to explore self-reported symptom occurrence and distress among Danish liver transplant recipients, and their association with self-reported psychological well-being, demographic, and clinical characteristics.

## Patients and methods

This nationwide cross-sectional study adhered to the STROBE guidelines for reporting observational studies (von Elm et al., 2014). The study was conducted at the Copenhagen Transplant Unit, which is the only unit in Denmark where LT is performed. Approximately, 50–60 liver transplantations are performed annually there (Scandiatransplant, 2024). After the early post-transplantation phase (>3 months), liver transplant recipients are offered lifelong follow-up care at four different centers in Denmark: Copenhagen, Odense, Aarhus, and Aalborg University Hospitals.

### Participants

Eligibility criteria: undergoing LT from 1990 to 2022, ≥ 18 years old at the time of inclusion, and Danish speaking. Exclusion criteria; unable to understand written Danish. We invited 511 persons who had undergone LT. The liver transplant recipients were identified through the Danish Comorbidity in Liver Transplant Recipients study (DACOLT) database and contacted by secure e-mail with information about the study, informed consent, and a personal link (Thomsen et al., 2021). DACOLT includes 85% of the Danish liver transplanted population.

### Data collection

Data was collected in January and February 2023. Demographic characteristics regarding age at data collection, gender, cohabiting status, education, employment, and clinical variables were retrieved from medical records and self-reports from the DACOLT study (Thomsen et al., 2021). Data on self-reported symptom distress, and psychological well-being was collected by means of three questionnaires. Symptom distress is defined as the recipient’s experience of the severity of the potential symptom, i.e., the impact on the persons everyday life. Psychological well-being is defined as the level of distress or absence of distress experienced by the patient.

### Symptom distress

To assess recipients’ self-reported symptom distress, the Organ Transplant Symptom and Well-being Instrument (OTSWI) was used (Forsberg et al., 2012). This instrument includes 20 single items measuring symptom distress by the extent of agitation from 20 transplant-specific symptoms on the following five-point scale: “not at all” (0), “a little” (Moayed et al., 2018), “somewhat” (World Health Organization, 2002), “quite a bit” (Ko et al., 2019), and “very much” (Vanhoof et al., 2018). Time is specified as a timeframe of 7 days. The most distressing symptoms are defined as “quite a bit” (Ko et al., 2019) and “very much” (Vanhoof et al., 2018).

### Symptom occurrence

The OTSWI also includes a questionnaire of eight transplant-specific dimensions developed to measure symptom prevalence, symptom distress, and level of well-being: fatigue (three items), joint and muscle pain (three items), cognitive functioning (two items), basic activities in daily life (three items), sleep problems (three items), mood (two items), foot pain (two items), and financial situation (two items) using the same response alternatives as described above. These factors form a total score from a minimum of 0 to a maximum of 80 points, where lower scores indicate higher transplant-specific well-being (Forsberg et al., 2012). A psychometric evaluation supports the internal consistency, reliability, and concurrent validity of the OTSWI for measuring symptom distress and well-being in relation to organ transplantation (Forsberg et al., 2012). The eight dimensions are added up and presented in percentages in relation to recipients who scored the items as “quite a bit” (Ko et al., 2019) and “very much” (Vanhoof et al., 2018). The two OTSWI questionnaires have a Cronbach’s alpha ranging from 0.81 to 0.92, respectively (Forsberg et al., 2012). The Cronbach’s alpha for the Symptom distress scale divided in eight dimensions are as follows: Fatigue (0.90), Joint and Muscle Pain (0.87), Cognitive function (0.84), Basic ADL (0.92), Sleeping problems (0.89), Mood (0.82), Foot pain (0.81) and Economy (0.81) (Forsberg et al., 2012).

### Psychological well-being

To assess the recipients self-reported psychological well-being, we used a Danish version of The Psychological General Well-being (PGWB) instrument (Wiklund and Karlberg, 1991). The instrument comprises 22 items that constitute six subscales, which are calculated to yield the total score. Higher scores indicate better health status and psychological well-being. The subscales include anxiety, depression, self-control, positive well-being, general health, and vitality. A total score of below 100 indicates poor PGWB, while a score above 100 suggests high PGWB. The highest possible score is 132, descending to 22 as the lowest possible score (Dimenas et al., 1996). The PGWB has an inter-item correlation value ranging from 0.52 to 0.79 and a Cronbach’s alpha ranging from 0.61 to 0.89 (Wiklund and Karlberg, 1991).

All questionnaires were translated forward and backward by professional translators and pilot-tested with patients for the specific purpose of the present study. The OTSWI was translated from Swedish into Danish, and the PGWB from English into Danish. None of the questionnaires had previously been used in Danish solid organ recipients.

### Statistics

Data was presented as percentages for categorical variables, and medians with interquartile range (IQR) for continuous data. The Chi-square and Fisher exact test were used to test differences in categorical variables. For continues variables the distribution of data has been investigated using Shapiro–Wilk normality test. For continuous variables, the Mann–Whitney U test was employed to test differences in two unpaired groups, while the Kruskal-Wallis test was applied to test differences in >2 groups. Spearman’s rho explored correlations between two continuous variables. For Symptom distress the dependent variable assessed were different symptoms (e.g., “feeling bloated”) with a distress score of at least 3 (“quite a bit”). These distressing symptoms were tested for sex differences (e.g., feeling bloated ~ sex). Furthermore, the symptom “decreased libido” was tested for differences in pre-vs. post-menopausal women (e.g., decreased libido ~ in pre-vs. post-menopausal status).

To explore post-transplantation PGWB, we divided the PGWB instrument into timeframes of ≤5 years, 5–10 years, 10–15 years,15–20 years and > 20 years after LT. For PGWB, the dependent variable assessed was the PGWB score. This score was assessed for differences in sex, age, level of education, years after transplantation (both as continuous and categorical) and number of transplant-specific symptoms (e.g., PGWB score ~ sex). The PGWB score was not normally distributed (Shapiro–Wilk tests, *p* = 7.3^−8^) and therefore non-parametric analysis was used.

A *p*-value <0.05 (two-tailed) was considered significant and 95% confidence intervals (95% CI) were included. All analyses were conducted in/using the R version 3.6.1 statistical software.

### Research ethics

DACOLT has been approved by the Committee on Health Research Ethics of the Capital Region of Denmark (approval number for DACOLT H-20052199). Written informed consent was obtained from all participants. The studies were conducted according to the Declaration of Helsinki (Thomsen et al., 2021).

## Results

### Characteristics of liver transplant recipients

The overall response rate was 46.6% (*n* = 238). Fifty two percent of invited male recipients and 39.7% of the invited female recipients responded. The recipients had been followed for a median of 7.9 years (IQR 3.9; 14.8 years) since LT and their median age was 59.8 years (IQR 50.6; 67.3 years). Forty-nine percent were women, and 24% of the recipients were living alone. Demographic and clinical characteristics are presented in Table 1.

**Table 1:** Sociodemographic and clinical characteristics of 238 Danish liver transplant recipients from year 1990–2021.

Gender	% Female (*n*)	48.7 (116)
Age	Median (IQR)	59.8 (50.6–67.3)
Living alone	% (*n*)	24.4 (58)
*Missing*	% (*n*)	2 (4)
Education		
*No education or short (max 3 years)*	% (*n*)	16.4 (39)
*Vocational*	% (*n*)	34.9 (83)
*College (3 years)*	% (*n*)	28.6 (68)
*University*	% (*n*)	18.9 (45)
*Missing*	% (*n*)	1 (3)
Work		
*Retired*	% (*n*)	35.7 (85)
*Missing*	% (*n*)	5 (11)
Physical activity		
*Little (less than 2 h a week of easy activity)*	% (*n*)	8.4 (20)
*Low (between 2–4 h a week of easy activity)*	% (*n*)	43.7 (104)
*Medium (> 4 h a week of easy activity)*	% (*n*)	36.6 (87)
*A lot (> 4 h a week of hard activity)*	% (*n*)	8.0 (19)
*Missing*	% (*n*)	3 (8)
Smoking		
*Current smokers*	% (*n*)	9.7 (23)
*Previous smokers*	% (*n*)	34.4 (82)
*Missing*	% (*n*)	1 (2)
Alcohol (daily or several times a week)	% (*n*)	13.8 (33)
*Missing*	% (*n*)	0 (0)
Menopause (females only)	% (*n*)	53 (62)
*Missing*	% (*n*)	1 (3)
Time since transplantation	Median (IQR)	7.5 (3.6–14.2)
Reason for transplantation		
Hepatocellular Cancer	% (*n*)	7.1 (17)
Autoimmune diseases	% (*n*)	42 (104)
Cirrhosis (Alcoholic or Cytogenic)	% (*n*)	17.6 (42)
Acute liver failure	% (*n*)	5.0 (12)
Metabolic liver disease	% (*n*)	3.8 (9)
Other	% (*n*)	23 (54)
*Missing*	% (*n*)	11 (25)
Meld score	Median (IQR)	13 (9.25–17.00)
*Missing*	% (*n*)	51 (120)
*Immunusuppresive treatment*		
Tacrolimus	% (*n*)	76.1 (181)
*Missing*	% (*n*)	8.8 (21)
Everolimus	% (*n*)	10.9 (26)
*Missing*	% (*n*)	6.7 (16)
Ciclosporin	% (*n*)	9.7 (23)
*Missing*	% (*n*)	5.9 (14)
Prednisolone	% (*n*)	49.2 (117)
*Missing*	% (*n*)	4.2 (10)

### Symptom distress

**Figure 1:**
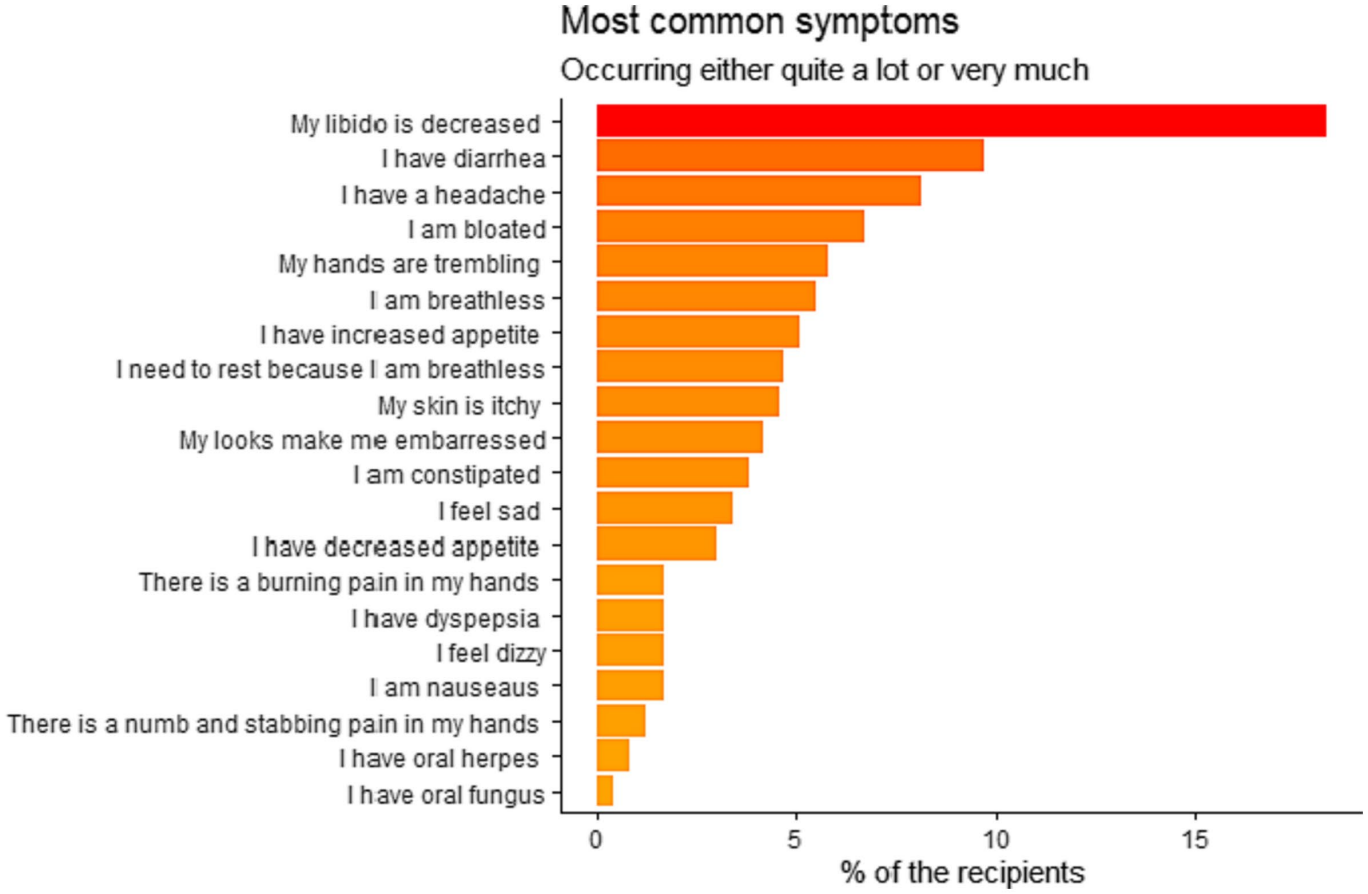
Most common distressing symptoms in Danish liver transplanted recipients (*n* = 238) who underwent liver transplantation from 1990 to 2021.

The most common single distressing symptom (18%) was decreased libido. The second and third most distressing symptoms were diarrhea (10%) and headache (8%). Further details are presented in Figure 1. There was no significant difference between postmenopausal and pre-menopausal women in terms of decreased libido (25% vs. 12%) (*p* = 0.257).

**Figure 2:**
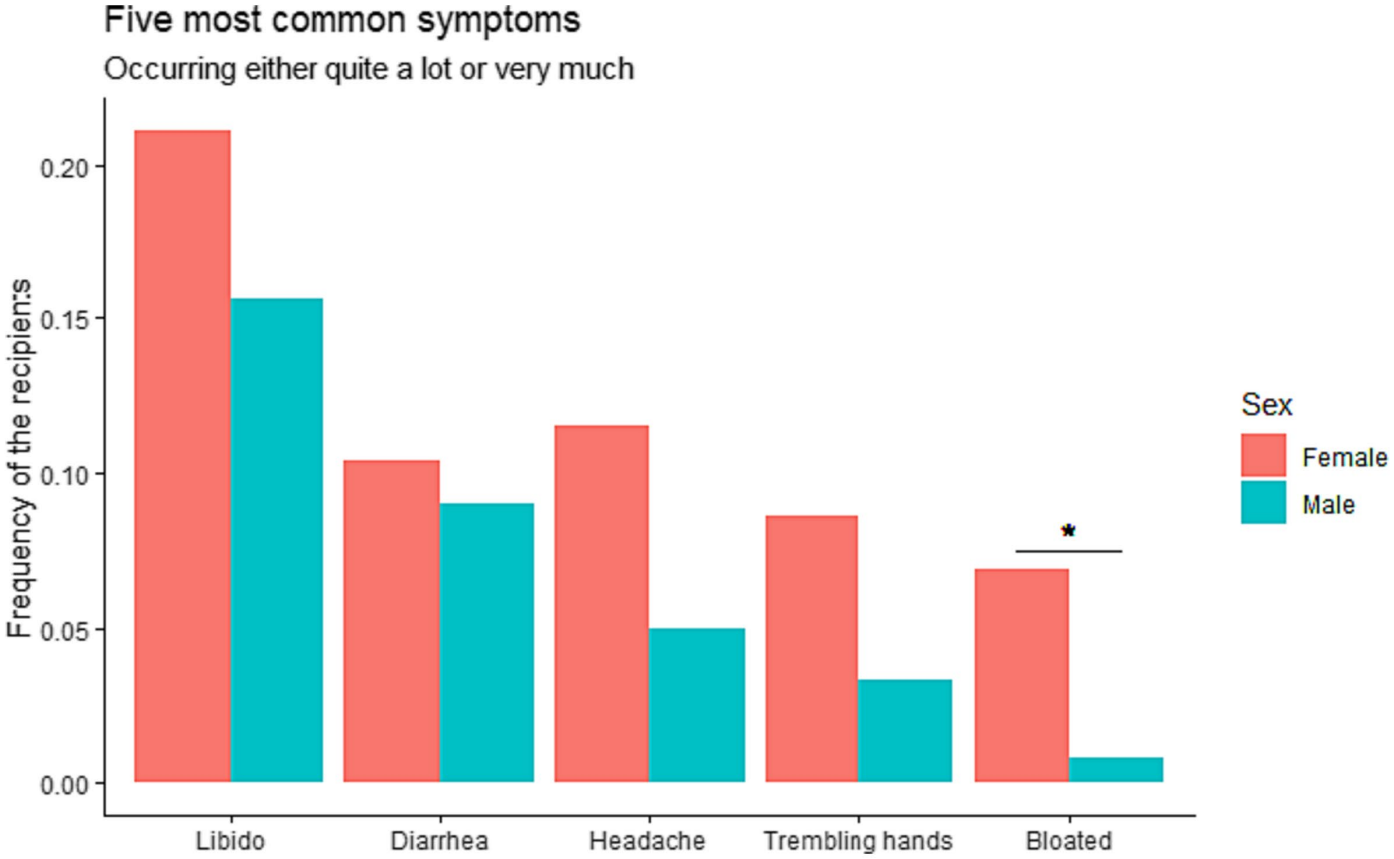
Gender differences in symptoms in Danish liver transplanted recipients (*n* = 238) transplanted 1990–2021 exhibiting the five most common symptoms, i.e., decreased libido, diarrhea, headache, trembling hands, and feeling bloated.

When exploring possible gender differences, there was a general tendency toward higher symptom distress among women. This difference was significant regarding “feeling bloated,” where women reported more symptoms than men (6.9% vs. 0.8%) (*p* = 0.0168). Further details are presented in Figure 2.

In relation to the association between the most common five symptoms and the reason for LT, significantly more recipients reported having headache in the autoimmune disease compared to the other reasons for LT: 14.7% of recipients transplanted for autoimmune diseases vs. 0, 5.7, and 2.5% for cancer, cirrhosis and other reasons, respectively (*p* = 0.0137). There were no differences for the other four most common symptoms (*p*-values not shown).

### Symptom occurrence

Symptom occurrences are visualized in Table 2.

**Table 2:** Symptom occurrence of 238 Danish liver transplant recipients from year 1990–2021.

Gobal score	Median (IQR)	16 (9, 24)		Scores of “quite a bit” or “very much”
Sleep problems	Median (IQR)	1.5 (1,2)	*n* (%)	91 (38)
Joint and muscle pain	Median (IQR)	1 (0,2)	*n* (%)	56 (24)
Foot pain	Median (IQR)	0 (0,1)	*n* (%)	24 (10)
Fatigue	Median (IQR)	1 (0.5, 2)	*n* (%)	56 (24)
Impaired cognitive functioning	Median (IQR)	1 (0, 1.5)	*n* (%)	33 (14)
Reduced	Median (IQR)	0 (0)	*n* (%)	6 (2.5)
Mood problems	Median (IQR)	0.5 (0, 1)	*n* (%)	13 (5)
Some level of worry about their financial situation	Median (IQR)	0 (0,1)	*n* (%)	37 (16)

### Psychological general well-being

**Figure 3:**
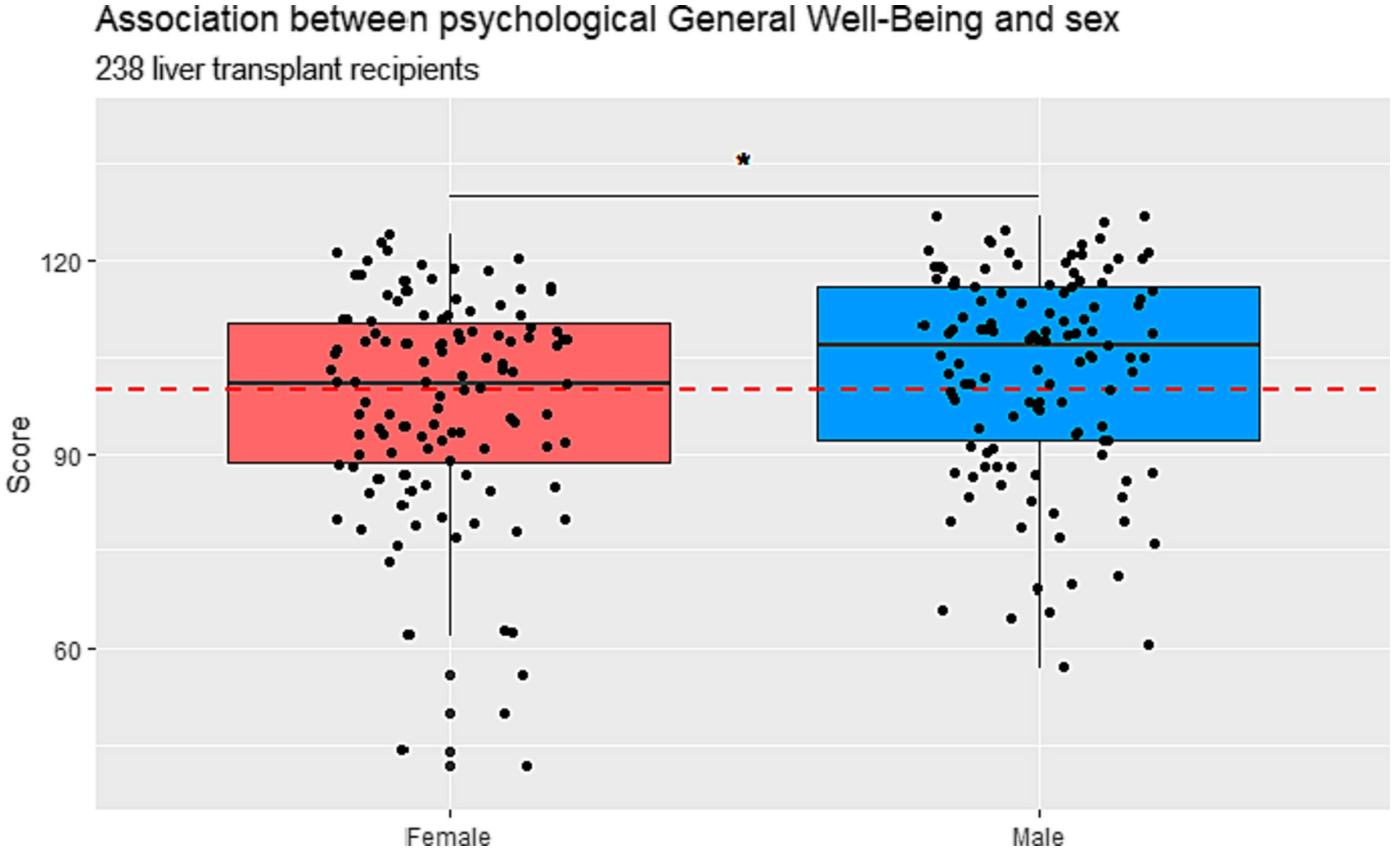
Psychological general well-being divided into male and female in Danish liver transplanted recipients (*n* = 238) who underwent liver transplantation in the years 1990–2021. Scores higher than 100 points indicate good psychological well-being and scores below 100 indicate poor psychological well-being.

The total median PGWB score was 104 (min-max 42–127, IQR 90–113), suggesting an overall intermediate psychological well-being among the recipients. 59% reported a score > 100 indicating good psychological well-being, while 41% scored <100, suggesting poor psychological well-being. Women had a significantly lower median score compared to men, 101 (IQR 89–110) in women vs. 107 (IQR 92–116) in men (*p* = 0.03). Further details are presented in Figure 3.

**Figure 4:**
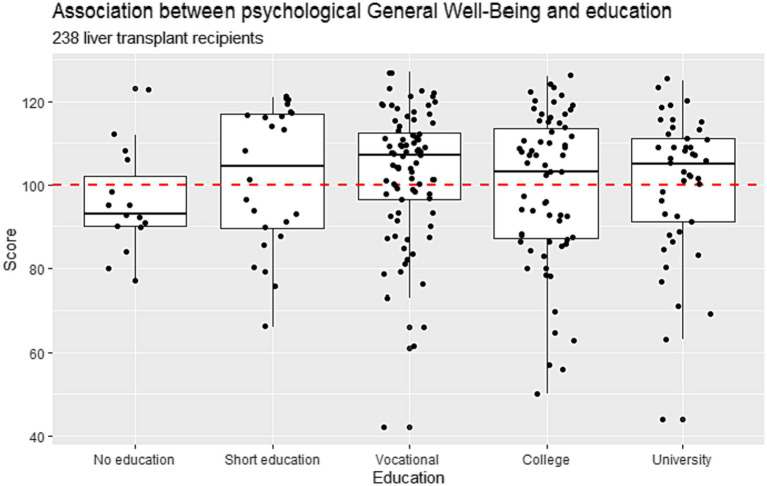
Association between psychological general well-being and levels of education: No, short, vocational, median, and long education in Danish liver transplanted recipients (*n* = 238) who underwent liver transplantation from 1990–2021.

There was a significant correlation between better psychological well-being and older age (Spearman’s rho 0.146) (*p* = 0.02). The correlation between PGWB and age-stratified by gender is visualized in Supplementary Figure S1. There was no association between the PGWB scores and different levels of education (*p* = 0.34). Further details are provided in Figure 4.

There was a weak but a significant correlation between higher reported PGWB scores and an increased number of years after LT (Spearmans rho = 0.137) (*p* = 0.03). Further details are presented in Supplementary Figure S2.

**Figure 5:**
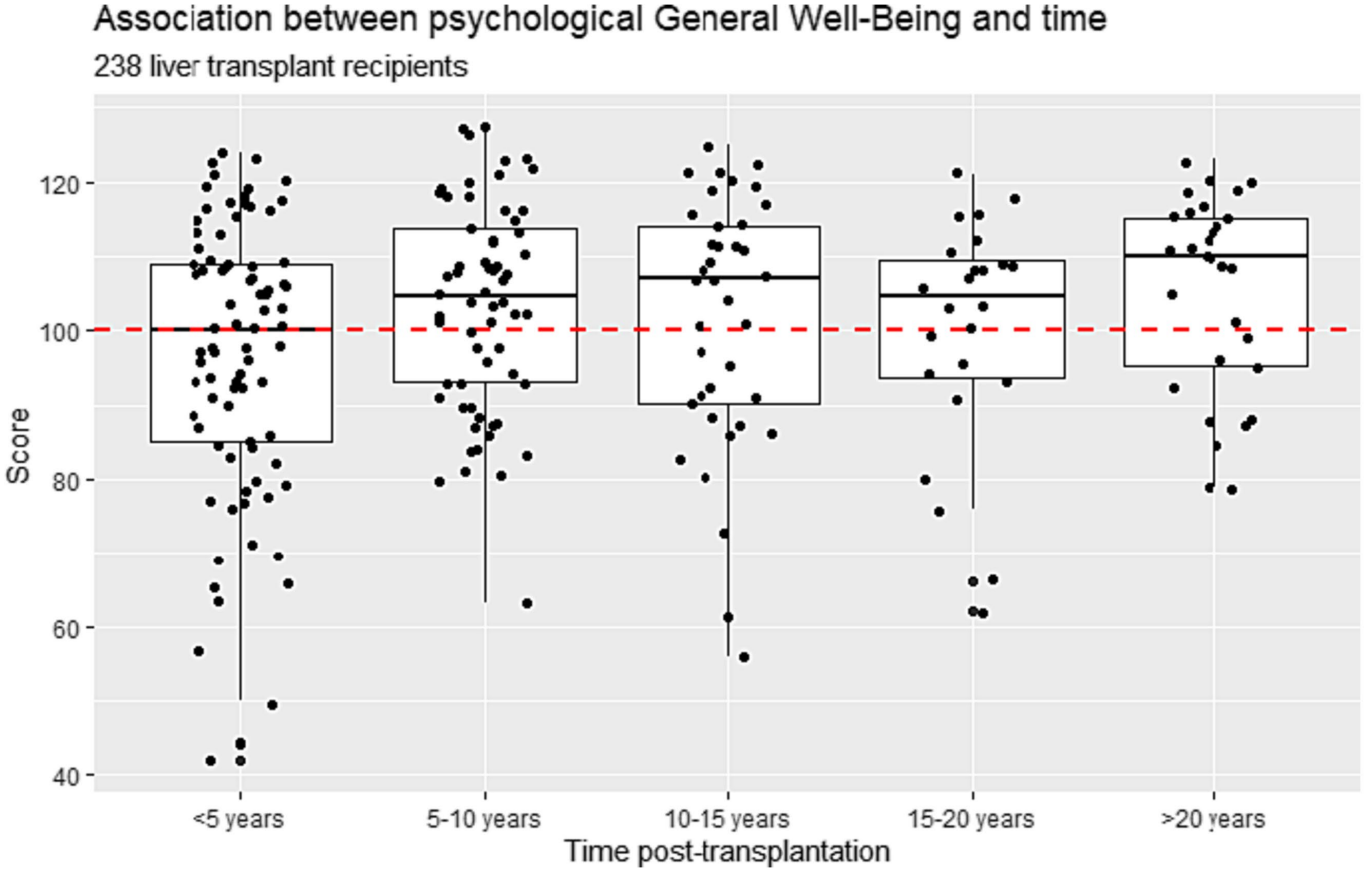
Association between psychological well-being and spans of time since transplantation divided into five-year periods in Danish liver transplanted recipients (*n* = 238) transplanted in 1990–2021.

49% reported poor PGWB in the first 5 years after LT (median 100, IRQ 85–109). After the first 5 years, the percentage of recipients reporting poor PGWB was 36% (median score 107, IQR 92–114). The PGWB scores in all four post-transplantation groups (5–10, 10–15, 15–20, and > 20 years after LT) increased. Further details are provided in Figure 5. Supplementary Figure S3 illustrates psychological well-being divided into time spans and recipients’ ages.

**Figure 6:**
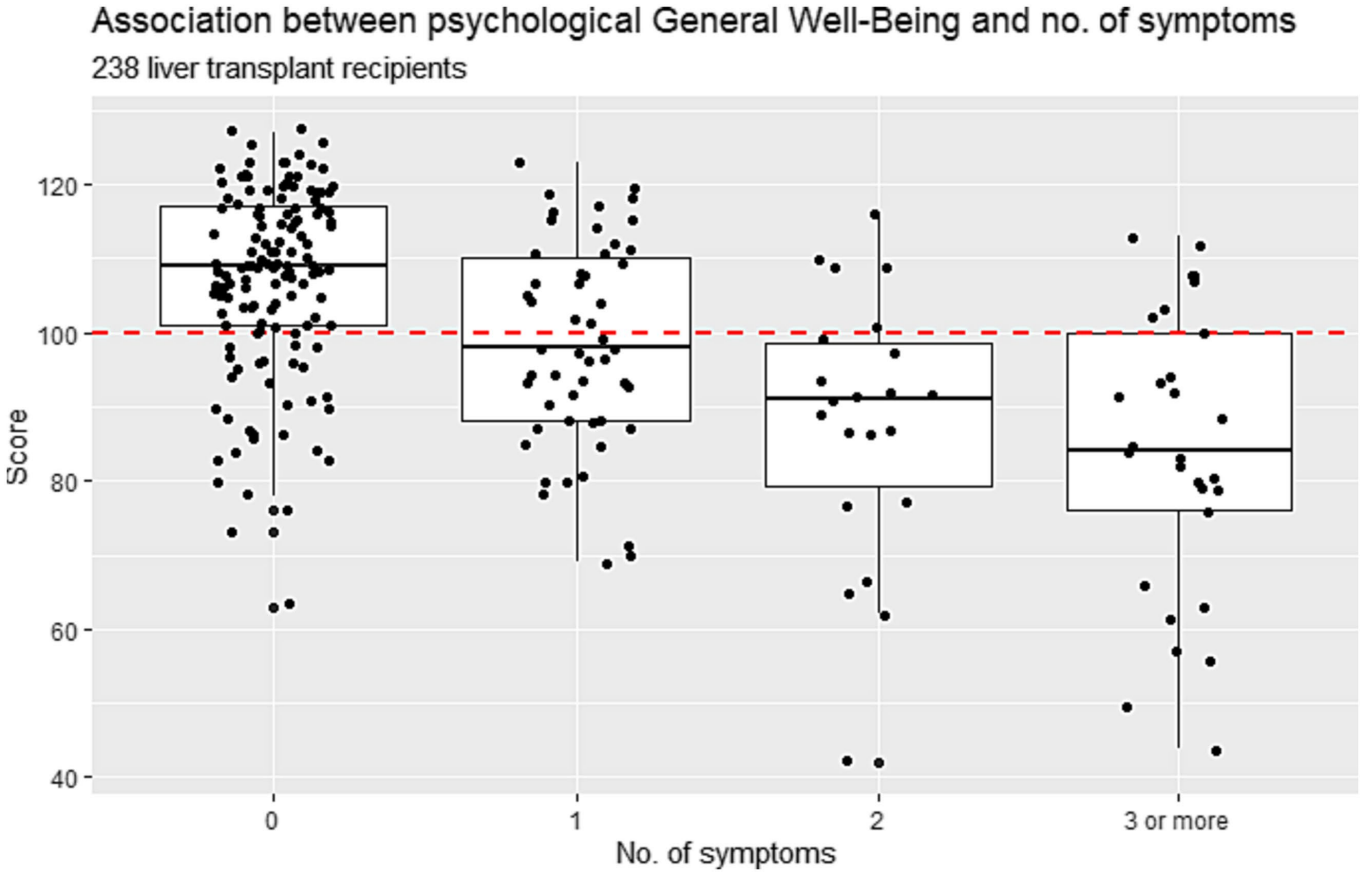
Association between psychological general well-being and number of transplant specific symptoms (0, 1, 2, 3) in Danish liver transplanted recipients (*n* = 238) who underwent liver transplantation from 1990–2021.

The PGWB score was strongly associated with the number of transplant-specific symptoms (*p* ≤ 0.001), with recipients experiencing more symptoms reporting a poor PGWB score. Recipients with no symptoms reported a median PGWB score of 109 (IQR 101–117), while those with three or more symptoms scored a median of 84 (IQR 76–100). Further details are provided in Figure 6.

There was no association between PGWB scores and recipients living alone or not living alone. Recipients living alone reported a median score of 106.5 (IQR 88.5–113.8) and recipients not living alone reported a median PGWB score of 103.5 (IQR 91–113) (*p* = 0.874). There was also no association between PGWB and steroid treatment as recipients treated with steroids reported a median PGWB score of 105 (IQR 88–112) and recipients not treated with steroids reported a median score of 103 (IQR 91.5–114) (*p* = 0.774).

## Discussion

This nationwide descriptive cross-sectional study provides a comprehensive picture of symptom distress and psychological well-being in a long-term perspective after LT. The key findings were that the most common symptoms reported by the recipients namely decreased libido, followed by diarrhea, and headache, were strongly related to poor psychological well-being. 41% of the recipients reported having poor psychological well-being, especially those transplanted within the previous 5 years. Recipients transplanted more than 5 years ago reported better psychological well-being than those who had undergone transplantation within the last 5 years, suggesting that liver recipients might reach a state of adaptation. Consequently, there should be a focus on symptom distress not only in the short-term perspective, i.e., the first 12 months, but for at least 5 years. After the first 5 years recipients have probably managed to adapt to most physical problems but not to decreased libido.

Decreased libido was the most common symptom in both genders with around 20% of the recipients scoring this condition as distressing. This finding is in line with a cross-sectional study from 2006 in which 26% of liver transplant recipients experienced decreased libido more than 1 year after transplantation (Ho et al., 2006). Studies investigating symptoms after lung (Lundmark et al., 2019) and heart transplantation (Dalvindt et al., 2020) also found decreased libido as the most prominent symptom. In the long term, the experience of decreased libido is a major issue that negatively affects the recipients’ QoL (Ho et al., 2006) and might also be related to depression (Baranyi et al., 2012). Therefore, it seems important that clinicians focus systematically on this concern during follow-up care and invite a sexologist to the transplant team for consultation. We need to decrease the stigma surrounding sexuality and decreased libido and include this area of people’s lives as a target for health promotion interventions. A higher number of post-menopausal women reported decreased libido, however the difference did not reach significance. This finding is reasonable, as the menopause and post-menopausal period include well-known hormonal changes that might affect libido. Even though older women are more likely to experience sexual dysfunction (Blanch et al., 2004), the specific components of sexual dysfunction after LT remain unexplored (Ho et al., 2006). The finding that decreased libido was the most prominent symptom might reflect the fact that recipients adjust to the rest of their symptoms but fail to adjust to decreased libido, making it a concern in the long-term perspective. Since libido and sexuality are a basic human need linked to QoL, we should pay more attention to barriers to a healthy sex life after transplantation (Ho et al., 2006; Onghena et al., 2016).

Gender differences were apparent regarding symptom distress and psychological well-being, where women reported more symptom distress and lower psychological well-being than men. These findings are in line with previous studies (Jalowiec et al., 2011; Nilsson et al., 2013; Forsberg et al., 2017, 2018; Lundmark et al., 2019; Almgren et al., 2021) and confirm the importance of considering a gender-diversified follow-up. For example, a recent study (Lundmark et al., 2019) found that women who had undergone lung transplantation reported worse joint and muscle pain, more sleep problems, and worse headache, nausea, and dizziness than men, who by contrast reported more trembling hands. Gender differences were also recent identified by in a study (Nilsson et al., 2013), who investigated the types of coping used to handle the threat of graft rejection among organ transplant recipients. They found that when compared to men, women tended to use more fatalism in their coping strategy in relation to graft rejection (Nilsson et al., 2013). A more comprehensive understanding of gender differences in the symptom experience after transplantation is needed as well as interventions focusing on how to accommodate these differences into the mandatory lifelong follow-up care. As the female patients in our study might be in the peri or menopausal age this might have affected our results (Santoro et al., 2021). However, our data did not provide the possibility to explore this, but a future prospective study would shed more light on this specific and important matter.

Almost half of the recipients reported good psychological well-being, which might be due to their ability to adapt and cope with their new normality. The downside is that half of the recipients had poor psychological scores, which highlights the need for increased psychological support and warrants the inclusion of a minimum of at least one psychologist or psychotherapist in the transplant team. It is known that steroid medication affects both physical and psychic presentation (Drent et al., 2008, 2009; Kugler et al., 2009). In our study, we found almost haft of the recipients were treated with steroids although this was not associated with the PGWB scores. However, a recent study by Lieber et al. (2022) found that recipients described the time after LT as an emotional roller coaster involving fear of not returning to normal, social isolation, identity issues, and loss of autonomy. All these concerns might be difficult to deal with alone and in our study may be reflected by the poor psychological well-being scores. As argued by Almgren et al. (2017b) symptoms, setbacks and complications cause uncertainty that leads to disappointments, grief, low self-efficacy, and psychological distress. The LTRs with low PGWB should be viewed as particularly vulnerable. They may be in great uncertainty due to their symptom distress and in need of targeted interventions with symptom management support, where a primary contact nurse could establish a continuous person centered caring relationship to enable personalized follow-up. As short-term survival after LT has improved extensively over recent decades, we need to focus on the long-term outcome (Fuochi et al., 2023). Even though LT is a lifesaving treatment it is a chronic condition where one moves from end-stage disease to hopefully better health and well-being. It is well known that lifelong immunosuppressive medication produce several side effects and possible serious complications. A person-centered approach where liver transplanted recipients’ understanding of and ability to cope with symptom distress are in focus should be the key component of symptom management support. When screening symptoms, signs of depression should also be included because depression is a predictor of mortality and morbidity (Dew et al., 2015).

Facing complications and disappointments is a reality for many transplant recipients and expectations need to be balanced to lower the risk of great disappointments on the one hand and of being inefficient and too reliant on external factors such as the healthcare system on the other. In our study we found no association between living alone or not and psychological well-being. As it is well known that living alone might have negative psychological consequences (Wu et al., 2022), our findings highlight the need for targeted individual follow-up care and screening for psychological symptoms.

The present study highlights the need for systematic screening of physical and psychological symptoms in recipients during mandatory follow-up care. Further implications include attention to the fact that it takes years for the recipients to adapt to the new situation. Systematic assessment of symptom distress should be mandatory during the first 5 years of follow-up. Furthermore, as there were many outliers with a range of symptoms the follow-up must be individualized so that recipients experiencing many symptoms receive more targeted follow-up care. Based on these findings more gender-diversified follow-up care should be considered in clinical practice, where health strategies and health promotion should be the key goal.

One of the main goals of transplantation is to improve the recipients’ QoL, while health promotion is the most important goal for transplant nursing. The findings from the present study highlight the fact that recovery can be complicated. We suggest that the long-term follow-up should be mainly organized as nurse-led follow-up. Transplant nurses should focus on how to assess and relieve symptom distress during follow-up and support adaptation to the new normality.

In conclusion, we found that the most commonly reported symptoms were decreased libido, diarrhea, and headache, and that 41% of the recipients reported poor psychological well-being. This was especially the case in recipients who had undergone transplantation within the last 5 years, women, and younger recipients. There was a strong relationship between symptom distress and psychological distress, thus the follow-up must include a thorough assessment of symptom distress and the transplant team should include at least one psychologist. Research focusing on symptom management with easy and rapid access to specialized healthcare professionals with interventions addressing both symptom distress and psychological well-being is warranted.

There are some limitations in this cross-sectional study. Firstly, the cross-sectional design limits the in-depth investigation of recipients’ perspectives and the causality of symptoms and well-being. In addition, there is a risk of selection bias, as non-responders may have differed in symptom distress, psychological well-being, or other variables. This might have skewed our results towards a lower prevalence of reported symptom distress and psychological well-being. Our study had few eligibility criteria, which strengthens the external validity of the findings. However, the results are best generalized to long-term recipients as the median time from LT was 7.9 years. A major strength of the study is the nationwide, multicenter design, and the use of the transplant-specific OTSWI with good psychometric properties (Forsberg et al., 2012) and the PGWB instrument with good internal consistency. Finally, this is the first study measuring symptom distress and psychological well-being in Danish liver transplanted recipients from a long-term perspective and as such is valuable.

## Data availability statement

The raw data supporting the conclusions of this article can not be made open according to Danish legislation. Requests regarding the data should be directed to the corresponding author.

## Ethics statement

The studies involving humans were approved by DACOLT has been approved by the Committee on Health Research Ethics of the Capital Region of Denmark (approval number for DACOLT H-20052199). Written informed consent was obtained from all participants. The studies were conducted according to the Declaration of Helsinki. The studies were conducted in accordance with the local legislation and institutional requirements. The participants provided their written informed consent to participate in this study.

## Author contributions

KD: Conceptualization, Data curation, Formal analysis, Funding acquisition, Investigation, Methodology, Project administration, Resources, Software, Supervision, Validation, Writing – original draft, Writing – review & editing. AK: Data curation, Investigation, Methodology, Software, Writing – review & editing. DM: Data curation, Formal analysis, Investigation, Methodology, Software, Writing – review & editing. AF: Conceptualization, Data curation, Formal analysis, Investigation, Methodology, Resources, Supervision, Writing – review & editing. SN: Conceptualization, Data curation, Formal analysis, Investigation, Methodology, Resources, Supervision, Writing – review & editing. JH: Resources, Supervision, Writing – review & editing.

## Supplementary material

Supplementary Figure S1 Correlation between Psychological General Well-being and age stratified by gender in Danish liver transplanted recipients (*n*=238) who underwent liver transplantation from 1990–2021.

Supplementary Figure S2 Correlation between Psychological General Well-being and increased number of years after liver transplantation in Danish liver transplanted recipients (*n*=238) who underwent liver transplantation from 1990–2021.

Supplementary Figure S3 Psychological General Well-being divided into time spans of five years and liver transplanted recipients’ age at time of liver transplantation in Danish recipients (*n*=238) who underwent liver transplantation from 1990–2021.
