# Supplementary figures and images for: Self-reported symptom occurrence and distress, and psychological well-being after liver transplantation – a descriptive cross-sectional study of Danish recipients
Source: Front Psychol. 2024 Mar 13;15:1354706. doi: 10.3389/fpsyg.2024.1354706 (PMC10965674; doi:10.3389/fpsyg.2024.1354706)

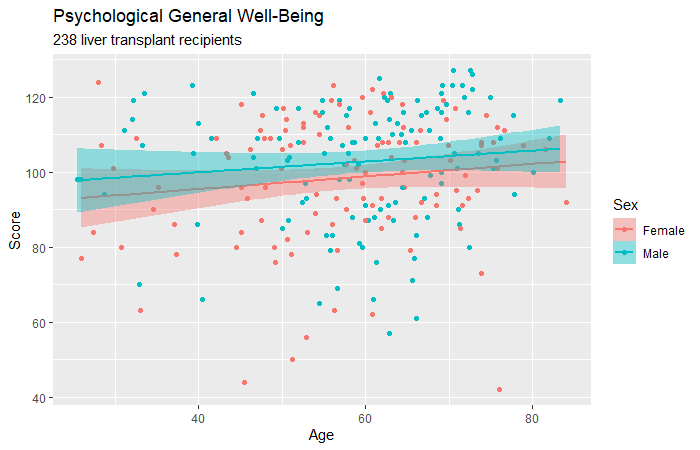

Supplement: Supplementary Figure S1 — Correlation between Psychological General Well-being and age stratified by gender in Danish liver transplanted recipients (n=238) who underwent liver transplantation from 1990–2021. [file Image_1.TIFF]

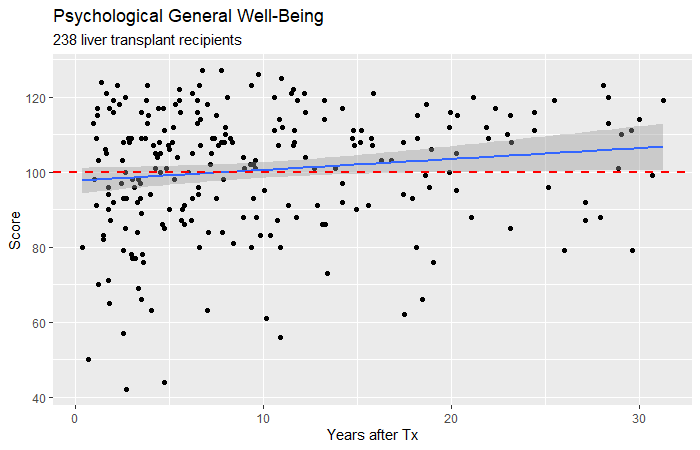

Supplement: Supplementary Figure S2 — Correlation between Psychological General Well-being and increased number of years after liver transplantation in Danish liver transplanted recipients (n=238) who underwent liver transplantation from 1990–2021. [file Image_2.TIFF]

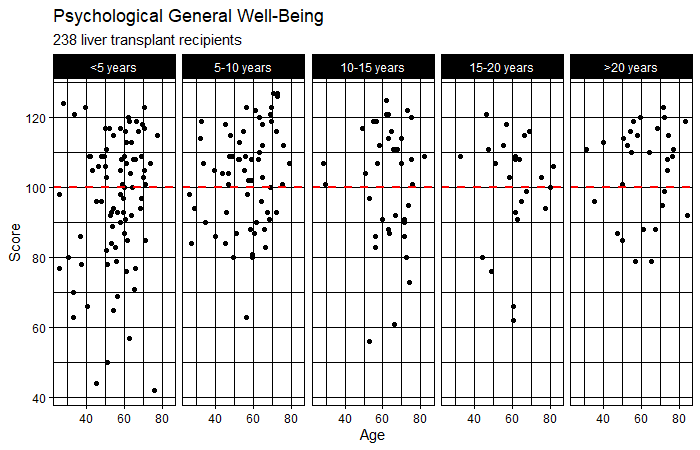

Supplement: Supplementary Figure S3 — Psychological General Well-being divided into time spans of five years and liver transplanted recipients’ age at time of liver transplantation in Danish recipients (n=238) who underwent liver transplantation from 1990–2021. [file Image_3.TIFF]
